# Lung function among children and adolescents with sickle cell disease living in Lake Victoria basin in Kenya

**DOI:** 10.3389/fped.2026.1792893

**Published:** 2026-06-09

**Authors:** Fredrick O. Olewe, Constance N. Tenge, Irene K. Marete, John M. Ong’echa, John G. Orimbo, Vincent Were, Godfrey A. Otieno, Bernhards R. Ogutu

**Affiliations:** 1 Department of Child Health and Pediatrics, School of Medicine, Moi University, Eldoret, Kenya; 2 Center for Research in Therapeutic Sciences (CREATES), Strathmore University, Nairobi, Kenya; 3 Centre for Global Health Research, Kenya Medical Research Institute, Kisumu, Kenya; 4 Centre for Clinical Research, Kenya Medical Research Institute, Kisumu, Kenya

**Keywords:** adolescents, children, Kenya, lung function, sickle cell disease

## Abstract

**Introduction:**

Sickle cell disease (SCD) is a group of inherited blood diseases in which the affected person inherits two abnormal haemoglobin genes from both parents and characterized by lifelong symptoms of anemia, vaso-occlusive crisis (VOCs) and end organ damage. Pulmonary complications that precipitate decline in lung function are contributors to morbidity and mortality. Determination of lung function can enable early detection of derangements allowing for timely institution of appropriate interventions to delay progression of symptoms.

**Aims:**

This study evaluated the lung function patterns and their determinants in persons aged 6–17 years with confirmed SCD diagnosis.

**Methods:**

One hundred and thirty-eight participants diagnosed with SCD attending care at Jaramogi Oginga Odinga Teaching and Referral Hospital in Kisumu, Kenya were recruited. Socio-demographic and clinical characteristics were obtained using a structured questionnaire and the lung function determined using spirometry (NDD Easy-On PC).

**Results:**

Abnormal lung function was noted in 27.5% (39/138) of which 66.7% (26/39) and 33.3% (13/39) were restrictive and obstructive type respectively with none being mixed. Residing in an urban setting [OR =  1.4026 (95% CI = 0.6508, 3.0230), *p* = 0.3878] underweight [1.9328 (0.5745, 6.5023) *p* = 0.2871], and using charcoal [1.3579 (0.6026, 3.0597 *p* = 0.4604] were not significantly associated with abnormal lung function, while, being on a stable hydroxyurea dose [0.8391 (0.3521, 1.9997) *p* = 0.6921] and hospital admission [0.9803 (0.4564, 2.1059) *p* = 0.9594] were negatively related.

**Conclusion:**

Therefore, abnormal lung function is common in young persons living with SCD in Lake Victoria Basin region thus, there is need for targeted lung function evaluation for appropriate and timely intervention.

## Introduction

Sickle cell disease (SCD) is a group of autosomal recessive inherited blood diseases in which the affected person inherits two abnormal Haemoglobin (Hb*)* genes from both parents and at least one of these abnormal genes must be the abnormal protein hemoglobin (Hb)S affecting the *β* hemoglobin chain of the short arm of chromosome 11 (1). The most common SCD abnormalities are HbSS (sickle cell anemia), HbSC, HbSD, HbSE, and HbS beta-thalassemia (2). In sub-Saharan Africa, approximately 240,000 children are born with the SCD each year and predominantly HBSS (3). Most of them die early without being diagnosed due to dearth of data on the pathogenesis and care programs which makes early identification and management difficult (4). SCD is responsible for 50%–80% of under-five mortalities in endemic countries (5).

According to Ministry of Health (MoH), 6,000 children in Kenya are born with SCD every year. In the absence of new-born screening and appropriate treatment, most of these children die undiagnosed in early childhood from preventable causes. The disease burden in Kenya is quite high especially around the lake regions as well as the Coastal regions. SCD remains the most common hemoglobinopathy encountered in Kenya where around 4.5% of children are born with SCD, and 18% with sickle cell trait (5, 6).

Children with homozygous state (Hb SS) usually have lifelong recurrent crises leading to acute and or chronic end organ damage presenting as renal failure, cerebrovascular incidents, chest syndrome, cardiomyopathy with stunting and underweight habitue (3, 5).

Progression and severity of the disease is influenced by factors such as genetic modifiers, nutritional status, and access to healthcare (7). Children with SCD continue to have pulmonary related morbidity and mortality, however, the burden and nature of abnormal respiratory function manifesting as obstructive or restrictive lung disease remains poorly understood yet it has been establish that adults progress to have restrictive pulmonary pathology (8, 9). Understanding of respiratory complications can be better achieved through targeted spirometry assessment of lung function, which is most readily available, non-invasive, and affordable.

In the USA it was noted that 30% of children with SCD had an abnormal lung function pattern with 16% and 7% being obstructive and restrictive respectively (10, 11) while in Uganda 37.9% children with SCD had abnormal lung function with 53.2% restrictive, 45.2% obstructive, and 1.6% mixed patterns (12). Risk factors for abnormal lung function in children with SCD include, history of acute chest syndrome (ACS), sex, malnutrition, Body Mass Index (BMI), hydroxyurea use, hospitalization and blood transfusions (13–16).

The current study determined the lung function and associated factors in young persons living with SCD in Kenya where there is paucity of data regarding the burden of abnormal lung function.

## Methods

This was a descriptive cross-sectional hospital-based study conducted at Jaramogi Oginga Odinga Teaching and Referral Hospital (JOOTRH) in Kisumu among young persons diagnosed with SCD. The participants age 6 to 17 years were recruited over a period of six months from 1st October 2023 to 30th March 2024. All parents/guardians and the adolescents provided consent and assent respectively. Patients with active respiratory infections or known diseases or unable to follow instructions during spirometry or using respiratory drugs were excluded. Data was collected using a predesigned questionnaire and medical information for the past 12 months was collected through interview and where there was concern, this was confirmed with the patient's records. Stable hydroxyurea dose was taken to be, a patient who was on appropriate weight based dose for the past 3 months as prescribe by the doctor and confirmed by the parent/guardian. ACS was taken to be history of chest pain with associated consolidation on x-ray and fever as documented by the clinician for which appropriate treatment was prescribed. VOC was taken to be history of non-self-limiting pain without any other possible explanation as diagnosed by the clinician.

Socioeconomic data was collected prospectively through the questionnaire (Supplementary Appendix I) completed by the study staff during the interview with the family member. The spirometry measurements were performed using NDD Easy-On PC. The study was approved by Institutional Research Ethics Committees (IREC) of Moi University/Moi Teaching and Referral Hospital and Jaramogi Oginga Odinga Teaching and Referral Hospital.

### Spirometry

Global Lung Initiative (GLI) 2012 reference equation for estimating the spirometry findings in black population were automatically selected from the spirometer before performing the test. These values were previously evaluated and validated in sub Saharan Africa (11, 17). The spirometry measurements were performed in triplicate to obtain the highest values of FEV _1,_ FVC, FEV_1_/FVC ratio, the predicted values and percent predicted values with minimal curve variability, and in conformity with standard international guidelines for European Respiratory Society and American Thoracic Society (Supplementary Appendix II) (16, 18). The results were interpreted as restrictive; FVC < 80% of predicted values, normal to high FEV_1_/FVC ratio >70%, obstructive; FEV_1_ < 80% or FEV1/FVC ratio <70% of predicted values, normal or lower FVC and mixed; FEV_1_ < 70% together with FVC < 80% predicted value (16, 18, 19).

Quality control was ensured by using the best values for interpretation in comparison to the predicted reference values with a margin of error of 0.05. The spirometry operators were trained and accredited by Respiratory Society of Kenya (RESOK).

### Data analysis

Data analysis was done according to the pre-determined statistical analysis plan (SAP) using STATA version 16.0 to compute the frequency and proportion for categorical data, mean, SD and range for continuous data. A bivariate analysis was done for association, odds ratio at 95% confidence level. Fishers exact tests where applicable was done to test for statistical significance.

## Results

A total of 151 participants were screened, 13 were excluded (8 had AS on Hb electrophoresis, 4 could not follow spirometry and one, the parent declined spirometry), thus 138 meeting the inclusion criteria and were considered for the final analyses.

### Patients' characteristics

Seventy-three (53%) participants were males, and the mean age, weight, height and BMI was 10.99 years 29.06 kg, 138.19 cm and 14.83 kg/m^2^ respectively (Table 1).

**Table 1:** Sociodemographic characteristics.

Demographics		Total *n* (%)	Abnormal lung function *n* (%)	Normal lung function *n* (%)
Sex	Male	73 (53)	18 (25)	55 (75)
	Female	65 (47)	20 (30)	45 (70)
Age	6–13	103 (75)	26 (25)	77 (75)
	>13–17	35 (25)	13 (37)	22 (63)
BMI	Normal	12 (9)	5 (42)	7 (58)
	Underweight	126 (91)	34 (27)	92 (73)
	Overweight	0 (0)	0 (0)	0 (0)
Area of residence	Rural	79 (57)	24 (30)	55 (70)
	Urban	59 (43)	14 (24)	45 (76)
Fuel used	Firewood	56 (41)	18 (32)	38 (68)
	Charcoal	58 (42)	15 (27)	43 (73)
	Gas	24 (17)	5 (21)	19 (79)
Cooking space	Indoor	125 (91)	33 (26)	92 (74)
	Outdoor	13 (9)	5 (39)	8 (61)

n, number; %, percentage.

### Clinical factors

A total of 102 (74%) of the participants were on stable dose of hydroxyurea within the last 3 months, 34 (25%) had no VOCs while 71 (51%) had 1–2 VOCs, 77 (56%) had no ACS while 52 (38%) had one to two ACS events in the preceding 12 months (Table 2).

**Table 2:** Clinical factors.

Clinical characteristics	Total *n* (%)	Abnormal lung function *n* (%)	Normal lung function *n* (%)
Stable hydroxyurea dose			
Yes	102 (74)	29 (28)	73 (72)
No	36 (26)	9 (25)	27 (75)
No. of VOCs within 12 months			
None	34 (25)	9 (27)	25 (73)
One-Two	71 (51)	20 (28)	51 (72)
>three	33 (24)	13 (39)	20 (61)
No. of ACS within 12 months			
None	77 (56)	20 (26)	57 (74)
One-Two	52 (38)	15 (29)	37 (71)
>three	9 (6)	3 (33)	6 (67)
No. of blood transfusion within 12 months			
None	82 (59)	22 (27)	60 (73)
One-two	46 (33)	13 (28)	33 (72)
>three	10 (4)	3 (30)	7 (70)
Hospital admission history within 12 months			
Yes	84 (61)	23 (27)	61 (73)
No	54 (39)	15 (28)	39 (72)

n, number; %, percentage.

Of the 138 children enrolled, 39 (28%) had abnormal lung function of which 26 (67%) and 13 (33%) being restrictive and obstructive lung disease respectively. There were no cases of mixed or Preserved Ratio Impaired Spirometry (PRISm) reported in our study.

### Factors associated with abnormal lung function

Residing in an urban setting [OR = 1.4.026 (95% CI = 0.6508, 3.0230), *p* = 0.3878] using charcoal [1.3579 (0.6026, 3.0597) *p* = 0.4604], and having had underweight [1.9328(0.5745, 6.5023) *p* = 0.2871] had positive, non-significant association with abnormal lung function, while being on a stable hydroxyurea dose [0.8391 (0.3521, 1.9997) *p* = 0.6921] and no hospital admission [0.9803 (0.4565, 2.1059) *p* = 0.9594 had a negative, non-significant association with abnormal lung function. In regards to the type of fuel, use of charcoal [1.3579 (0.6026, 3.0597) *p* = 0.4604] or gas [1.8000 (0.5794, 5.5922) *p* = 0.3095 had non-significant association with abnormal lung function. (Supplementary Table S1).

Neither the caregiver type nor level of education had significant association with abnormal lung function (*p* = 0.184 and 0.980 respectively) (Supplementary Table S2).

In terms of clinical factors and the patterns of abnormal lung function, participants who had received blood transfusion for more than 3 times within the preceding 12 months were significantly more likely to have abnormal lung function (*p* = 0.031) (Supplementary Table S3).

## Discussion

The study reports lung function abnormality in 28% of young persons living with SCD in Lake Victoria Basin in Kisumu of which 67% and 33% were restrictive and obstructive type respectively with being underweight, Using charcoal and living in urban setting being associated with minimal increased risk but statistically not significant. However, patients who had no hospital admissions and those on stable hydroxyurea dose were negatively associated with development of abnormal lung function though not statistically significant. The findings on prevalence of abnormal lung function are in keeping with reports in children in Nigeria (38%) and United Kingdom (UK) (27%) (20). The restrictive pattern type predominant in this study could be due to most of the children being underweight which is associated with restrictive lung function (13) as had previously been reported from Ghana and Uganda (11, 21). This is opposed to the obstructive type which is predominant in European based populations (22). These findings lend credence to why normal BMI was negatively related to occurrence of abnormal lung function even though it was not statistically significant. However, in a Caucasian study, high BMI was associated with abnormality majorly the obstructive type of abnormal lung function (15). Experiencing ≥ 2 episodes of ACS, VOC or transfusion episodes in the recent past 12 months did not result in a association with abnormal lung function and this contradicts earlier reports from Nigerian children (11, 13, 21). This could be due to differences in sample size and population of study.

We found that living in urban setup had an increased risk of having abnormal lung function which was not statistically significant compared to rural setup. This may be attributed to the industrial and motor vehicle emissions causing air pollution in urban centers (23) or those who have severe form of disease tend to live in urban areas where care is easily accessible as we tend to have a number of SCD patients in Kisumu being in day schools and staying with family members in the city (Ogutu B. *personal communication*). In this study being female was associated with decreased risk of having an abnormal lung function, different from the findings in Central Africa (13). However, this was in contrast to some previous studies which showed that sex does not impact lung function among children with SCD (23, 24). This may be explained by the African set up where older females dominate the cooking space which can predispose to respiratory pathology while men are majorly in the fields. The use of charcoal and gas compared to using firewood were found to increase the risk and this can be attributed to cooking in a confined environment as firewood is majorly used in outdoor space. This is in agreement with studies done in Nigeria where solid fuel use such as charcoal and firewood was associated with abnormal lung function with no statistical significant (20).

Certain factors, without being statistically significant, such as being on a stable dose of hydroxyurea in the recent 3 months, multiple blood transfusion as well as no hospitalization in the last one year being protective to developing abnormal lung function had also been previously reported in a large met-analysis study (25). The history of no hospitalizations being protective could be due to the fact that these patients present early with complications which are promptly treated as outpatient preventing development of abnormal lung functions. However, history of hospitalization effect was not statistically significant in South African children (26, 27) as is the case in this study. These variations could be attributed to population and study design differences. These findings give clear indication for the need of a robust clinical care for children with SCD ensuring access to health care in the developing worlds and in cooperating targeted lung function test as part of the package.

## Strengths and limitations

This study had the strength of being the first study in Kenya documenting the burden of abnormal lung function and the various patterns among children with SCD. It was limited by the small sample size hence there is need for longitudinal studies with larger sample sizes to confirm and or document any differences in spirometry findings. There is need for multi-center study in order to allow for external validity, even though the center used is a regional referral hospital limiting the selection bias. We also used only spirometry to measure lung function, which has limitations mostly when used in children and may require other modalities to confirm the results. The impact of using spirometry only was reduced by appropriate selection of the age group that could follow instructions.

## Conclusion

There is a high burden of abnormal lung function among children with SCD hence the need for targeted spirometry testing among these children to improve their care.

## Data Availability

The datasets presented in this article are not readily available because of local regulation requiring ethics approval before sharing with third parties. Requests to access the datasets should be directed to Institutional Research Ethic Committee of Moi University: Phone. +254787723677; Email. irecoffice@gmail.com or irec@mtrh.go.ke.
