# Supplementary material for: Lung function among children and adolescents with sickle cell disease living in Lake Victoria basin in Kenya
Source: Front Pediatr. 2026 Jun 9;14:1792893. doi: 10.3389/fped.2026.1792893 (PMC13286934; doi:10.3389/fped.2026.1792893)
Supplement: Supplementary file 1 [file Supplementaryfile1.docx]

# Supplemental materials

## 1.0: DATA COLLECTION TOOL

**INSTRUCTIONS FOR USE:**

- Fill in responses in the columns on the right
- Special instructions written in italics
- Check the boxes as appropriate

| Participants’ number: | __ __ __ __ |
| --- | --- |
| Date of interview: | _ _ /_ _/_ _ _ _ |
| Time of interview: | _ _:__ |
| Interviewers name and signature: |  |

1. **Respondents Relationship to Child:**

Father
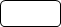


Mother
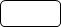


Guardian
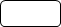


Others
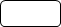


If others please specify________________________________________________

Level of education of the parent/guardian (***the person living with the participant***)

a)None
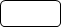
 b)Primary
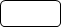
 c)Secondary
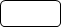
 d)Tertiary
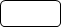


1. **Patient Characteristics**

**Socio-Demographic Data:**

1. Date of Birth: _________________ Age in years: ___________
2. Gender: a. Male b. Female
3. Residence: County_______________ Sub-County____________

Ward: __________________Village: ________________.

1. Type of residence:

a)Rural setup
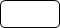
 b) urban setup.
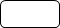


**5**. a)Type of fuel used at home _____________ b) Cooking place i) Indoor
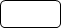
 ii) Out door
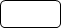


**6**. Weight: _______ Height: ________ BMI: _______ Percentile: _______

a) Normal
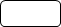
 b) Underweight
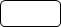
 c) Overweight
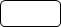


**Clinical Characteristics:**

**7**. No of Vaso-Occlusive Crisis (VOC) in the past 12 months

a) 0
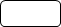
 b)1-2
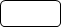
 c) >3
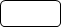


**8**. Number of Acute chest syndrome (ACS) episodes in the last 12 moths

a) 0
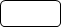
 b)1-2
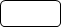
 c) > 3
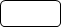


**9.** The number of blood transfusions received in the last 12 months

a) 0
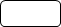
 b)1-2
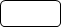
 c) >3
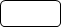


**10**. Is the patient on stable hydroxyurea dose for the last 3 months

a)Yes
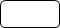
 b) No
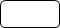


**11**. Is there known history of asthma?

a)Yes
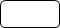
 b) No
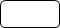


**12**. Does the patient have a history of wheezing?

a) Yes
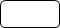
 b) No
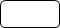


**13**. Is there any chest deformity noted by the clinician in this visit?

a)Yes
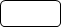
 b)No
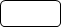


If yes, what is the diagnosis? ____________________

**14.** Any other Concurrent chronic illness? Yes
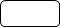
 No

If yes Specify______________________________________________

**15.** Any known congenital heart anomalies: ____________________________

**16**. Have you been admitted in the last 12 months**?** Yes a) No

**4. SPIROMETRY FINDINGS**

| **Parameters** | **Value** | **Predicted** | **Percent of predicted** |
| --- | --- | --- | --- |
| Forced Vital Capacity (FVC) |  |  |  |
| Forced expiratory volume in the first second (FEV_1_) |  |  |  |
| Ratio of forced expiratory volume in the first second to forced vital capacity FEV_1_/FVC |  |  |  |

1. **INTERPRETATION OF SPIROMETRY VALUE**
2.
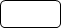

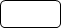

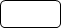
Restrictive 2. Obstructive 3. Mixed

4. PRIsm
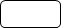


## 2.0 SUPPLEMENTAL TABLES

### 2.1 Supplemental Table 1: Factors associated with abnormal lung function


| **Associated Factors** |  | **Frequency (n)** | **Odds Ratio** | **Z statistics** | **P-value** | **[95% CI]** Lower, upper |
| --- | --- | --- | --- | --- | --- | --- |
| **Sex** | Male | 73 | REF |  |  |  |
|  | Female | 65 | 0.7364 | 0.801 | 0.4231 | 0.3482, 1.5570 |
| **Weight** | Underweight | 126 | 1.9328 | 1.065 | 0.2871 | 0.5745, 6.5023 |
|  | Normal | 12 | REF |  |  |  |
|  | Overweight | 0 | 0 |  |  |  |
| **Age in years** | 6-13 | 103 | REF |  |  |  |
|  | >13 | 35 | 0.5714 | 1.342 | 0.1795 | 0.22524, 1.2938 |
| **Type of residence** | Rural | 79 | REF |  |  |  |
|  | Urban | 59 | 1.4026 | 0.864 | 0.3878 | 0.6508, 3.0230 |
| **Type of fuel used at home** | Firewood | 56 | REF |  |  |  |
|  | Charcoal | 58 | 1.3579 | 0.738 | 0.4604 | 0.6026, 3.0597 |
|  | Gas | 24 | 1.8000 | 1.016 | 0.3095 | 0.5794, 5.5922 |
| **Cooking space** | Indoor | 125 | REF |  |  |  |
|  | Outdoor | 13 | 0.5739 | 0.918 | 0.3588 | 0.1753, 1.8790 |
| **No. of VOC in the past 12 months** | 0 | 34 | REF |  |  |  |
|  | 1-2 | 71 | 0.9180 | 0.182 | 0.8555 | 0.3655, 2.3054 |
|  | ≥ | 33 | 0.5538 | 1.121 | 0.2625 | 0.1970, 1.5568 |
| **No. of ACS in the past 12 months** | 0 | 77 | REF |  |  |  |
|  | 1-2 | 52 | 0.8655 | 0.360 | 0.7190 | 0.3940, 1.9014 |
|  | ≥3 | 9 | 0.8187 | 0.271 | 0.7862 | 0.1930, 3.4738 |

| **Associated Factors** |  | **Frequency (n)** | **Odds Ratio** | **Z statistics** | **P-value** | **[95% CI]**  **Lower, upper** |
| --- | --- | --- | --- | --- | --- | --- |
| **No. of blood transfusions received in the last 12 months** | 0 | 82 | REF |  |  |  |
|  | 1-2 | 46 | 0.9308 | 0.174 | 0.8616 | 0.4155, 2.0851 |
|  | ≥3 | 10 | 0.8556 | 0.213 | 0.8316 | 0.2031, 3.6040 |
| **Stable hydroxyurea dose for the last 3 months** | No | 36 | REF |  |  |  |
|  | Yes | 102 | 0.8391 | 0.396 | 0.6921 | 0.3521, 1.9997 |
| **Admission in the last 12 months** | No | 54 | 0.9803 | 0.051 | 0.9594 | 0.4564, 2.1059 |
|  | Yes | 84 | REF |  |  |  |

## 2.1 Supplemental Table 1: Factors associated with abnormal lung function

### 2.2 Supplemental Table 2. Association between caregiver characteristics and abnormal lung functions

|  | **Lung function** | | | |  | |
| --- | --- | --- | --- | --- | --- | --- |
| **Caregiver Characteristic** | **Normal n (%)** | | **Abnormal n (%)** | | P-value | |
| **Caregiver type** |  | |  | |  | |
| Father | | 8 (61.5) | | 5 (38.5) | |  |
| Mother | | 74 (77.9) | | 21 (22.1) | | 0.184 |
| Guardian | | 12 (92.3) | | 9 (7.7) | |  |
| Others | | 5 (55.6) | | 4 (44.4) | |  |
| **Caregiver education level** |  | |  | |  | |
| None | | 1 (33.3) | | 2 (66.7) | |  |
| Primary | | 24 (70.6) | | 10 (29.4) | | 0.098 |
| Secondary | | 39 (66.1) | | 20 (33.9) | |  |
| Tertiary | | 35 (83.3) | | 7 (16.7) | |  |

Statistically significant p value 0.05

# 2.3 Supplemental Table 3: Clinical factors and patterns of abnormal lung functions

| Factors |  | Restrictive  (Low FVC)  n (%) | Obstructive  (Low FEV_1_) n (%) | P-value |
| --- | --- | --- | --- | --- |
|  |  |  |  |  |
| No. of Vaso-Occlusive Crises | 0 |  |  |  |
|  | 1-2 | 7 (70) | 3 (30) |  |
|  | 3 | 13 (85) | 2 (15) | 0.614 |
|  |  |  |  |  |
| Number of Acute chest syndrome (ACS) | 0 | 11 (75) | 3 (25) |  |
|  | 1-2 | 16 (78) | 4 (22) |  |
|  | 3 | 12 (73) | 5 (27) | 0.816 |
|  |  |  |  |  |
| Number of blood transfusions received in the last 12 months | 0 | 2 (9) | 20 (91) |  |
|  | 1-2 | 10 (77) | 3 (23) |  |
|  | 3 | 2 (67) | 1 (33) | **0.031** |
|  |  |  |  |  |
| Patient on stable hydroxyurea dose for the last 3 months | Yes | 7 (78) | 2 (22) |  |
|  | No | 22 (76) | 7 (24) | 0.566 |
|  |  |  |  |  |
| History of asthma | Yes | 8 (73) | 3 (27) |  |
|  | No | 1 (100) | 0 (0) | 0.573 |
|  |  |  |  |  |
| History of wheezing | Yes | 29 (76) | 9 (24) |  |
|  | No | 3 (100) | 0 (0) | 0.314 |
|  |  |  |  |  |
| Admitted in the past 12 months | Yes | 14 (61) | 9 (39) |  |
|  | No | 10 (67) | 5 (33) | 0.434 |

n= frequency %=percentage

## RECRUITMENT FLOW CHART


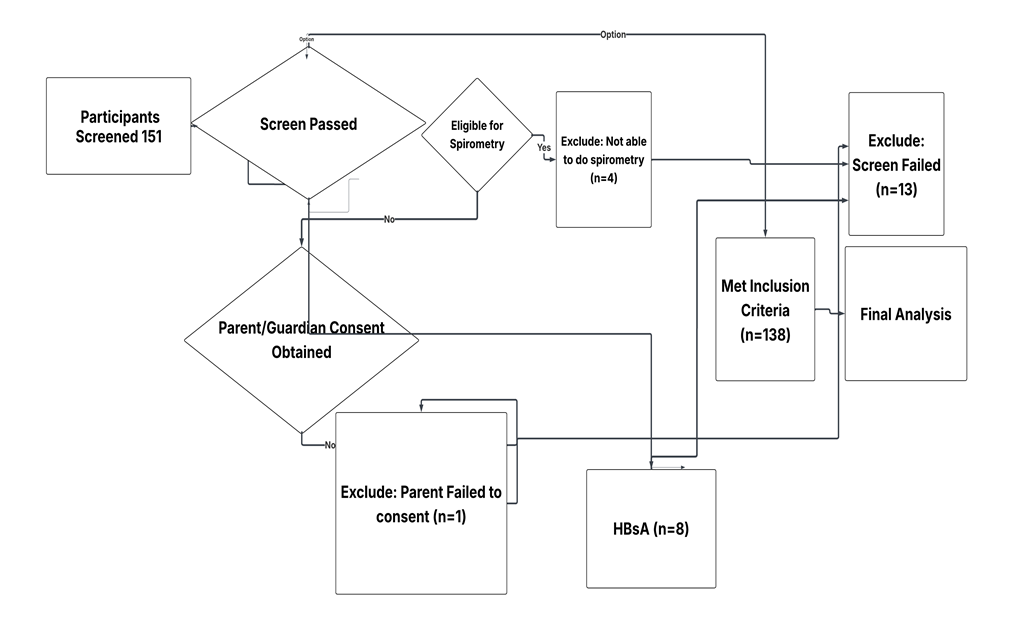


## 4.0 PROTOCOL FOR SPIROMETRY

**Maneuvers**

In order to perform spirometry that meets the requirement of ERS/ATS, the older child or adult must inspire to total lung capacity (TLC), exhale forcefully to residual volume (RV), and repeat the maneuver several times until reproducible flow–volume curves are evident of which the best three can be taken for analysis and interpretation. The repeatability of these curves is dependent on expiratory flow limitation which is defined as the flows being independent of effort (1). There are many ways that have been proposed for doing spirometry. However, the most common accepted globally is the ATS/ERS guidelines which were used in this study as suggested by Moore (2).

**Calibration**

Before performing spirometry, the equipment to be used must be calibrated according to the manufacturer’s instruction on a regular basis. In addition, the calibration should be checked at the beginning of every session to ensure credible results. Depending on the type of equipment, this can be achieved using either a 3-L syringe that is pumped through to check that the meter is reading correctly within a tolerance of 3% or using a 1-L syringe that is pumped repeatedly to a maximum of 7 L, this checks the linearity as well as the center point of the volume measurement. Some portable meters do not require calibration, for example those that use ultrasound technology (1).

**Prior to testing**

Prior to performing spirometry, the patient’s bio data should be checked and height, weight, age, sex and race recorded. For patients who are unable to stand in order to have their height measured, arm span can be used as an estimate. The patient should avoid drinking alcohol 4 hours before, eating large meal 2 hours before, avoid vigorous exercise 30 minutes before, smoking for more than 1 hour and document all medication used and time last taken (3).

**Contraindications**

The following are some contraindications for spirometry; hemoptysis of unknown origin, pneumothorax, unstable cardiovascular status, recent myocardial infarction or pulmonary embolism, thoracic, abdominal or cerebral aneurysms, a recent eye surgery, acute disorders affecting test performance, such as nausea or vomiting and recent thoracic or abdominal surgical procedures (4).

**Patient positioning**

During the procedure the patient should Sit or stand upright and there should be no restrictions. The feet should rest flat on floor with legs uncrossed, and all tight-fitting clothing should be loosened as this can give restrictive pictures on spirometry i.e. give lower volumes than are true. dentures can be left in; use a chair with arms to prevent the patient from swaying when exhaling maximally as they can become light-headed. It should be done in a very quiet place separate from the general waiting room (5).

**Infection control**

Hand washing should be done between patients; disposable mouth pieces should be used and thrown away at the end of testing. If an infectious patient requires testing, this should be performed last and equipment cleaned and sterilized (2).

**Technique**

There are a number of techniques which have been proposed for performing spirometry. Before performing the forced expiration, tidal (normal) breaths can be taken first, then a deep breath taken in while still using the mouthpiece, followed by a further quick, full inspiration. Alternatively, a deep breath can be taken in, then the mouth placed tightly around the mouthpiece before a full expiration is performed or the patient can be asked to completely empty their lungs then take in a quick full inspiration, followed by a full expiration (1).

**Quality**

A number of criteria for acceptable spirometry have been published. According to guidelines from the American Thoracic Society (ATS)/ European Respiratory Society (ERS) Task Force, an acceptable maneuver is defined as an explosive start (no hesitation or sigmoid curve) with a back-extrapolation volume of 150 mL. The maneuver should be performed with a maximal inspiration and expiration, no glottis closure or cessation of airflow occurred during the maneuver e.g. by hesitation or blocking the mouthpiece. There should be no coughs during the trace or evidence of leaks and finally the maneuver should meet the end-of-test criteria (exhaling for 06 s with 50 mL being exhaled in the last 2 s) (2).

##### References

1. Beydon N, Davis SD, Lombardi E, Allen JL, Arets HGM, Aurora P, et al. An Official American Thoracic Society/European Respiratory Society Statement: Pulmonary function testing in preschool children. Am J Respir Crit Care Med. 2007;175(12):1304–45.

2. Moore VC. Spirometry: Step by step. Breathe. 2012;8(3):233–40.

3. Arets HGM, Brackel HJL, Van Der Ent CK. Forced expiratory manoeuvres in children: Do they meet ATS and ERS criteria for spirometry? Eur Respir J. 2001;18(4):655–60.

4. Vogt B, Falkenberg C, Weiler N, Frerichs I. Pulmonary function testing in children and infants. Physiol Meas. 2014;35(3).

5. Graham BL, Steenbruggen I, Barjaktarevic IZ, Cooper BG, Hall GL, Hallstrand TS, et al. Standardization of spirometry 2019 update an official American Thoracic Society and European Respiratory Society technical statement. Am J Respir Crit Care Med. 2019;200(8):E70–88.
